# Promotion of Spinal Cord Regeneration by Neural Stem Cell-Secreted Trimerized Cell Adhesion Molecule L1

**DOI:** 10.1371/journal.pone.0046223

**Published:** 2012-09-25

**Authors:** Xiaowen He, Michael Knepper, Cheng Ding, Jun Li, Suita Castro, Maham Siddiqui, Melitta Schachner

**Affiliations:** 1 Keck Center for Collaborative Neuroscience and Department of Cell Biology and Neuroscience, Rutgers University, New Brunswick, New Jersey, United States of America; 2 Zentrum für Molekulare Neurobiologie, Universitätskrankenhaus Hamburg-Eppendorf, Universität Hamburg, Martinistr. Hamburg, Germany; 3 Center for Neuroscience, Shantou University Medical College, Shantou, P.R. China; VIB & Katholieke Universiteit Leuven, Belgium

## Abstract

The L1 cell adhesion molecule promotes neurite outgrowth and neuronal survival in homophilic and heterophilic interactions and enhances neurite outgrowth and neuronal survival homophilically, i.e. by self binding. We investigated whether exploitation of homophilic and possibly also heterophilic mechanisms of neural stem cells overexpressing the full-length transmembrane L1 and a secreted trimer engineered to express its extracellular domain would be more beneficial for functional recovery of the compression injured spinal cord of adult mice than stem cells overexpressing only full-length L1 or the parental, non-engineered cells. Here we report that stem cells expressing trimeric and full-length L1 are indeed more efficient in promoting locomotor recovery when compared to stem cells overexpressing only full-length L1 or the parental stem cells. The trimer expressing stem cells were also more efficient in reducing glial scar volume and expression of chondroitin sulfates and the chondroitin sulfate proteoglycan NG2. They were also more efficient in enhancing regrowth/sprouting and/or preservation of serotonergic axons, and remyelination and/or myelin sparing. Moreover, degeneration/dying back of corticospinal cord axons was prevented more by the trimer expressing stem cells. These results encourage the view that stem cells engineered to drive the beneficial functions of L1 via homophilic and heterophilic interactions are functionally optimized and may thus be of therapeutic value.

## Introduction

The adhesion molecule L1 has been shown to favour conducive processes in a generally inhibitory environment [1], [2], [3]. L1 is a member of the immunoglobulin superfamily and promotes neurite outgrowth in a homophilic (i. e. self-binding) manner [4]. *In vitro* and *in vivo* studies support the view that homophilic interactions of L1 not only promote neurite outgrowth and neuronal migration, but also neuronal survival [4], [5], [6], [7], [8], [9], [10]. Furthermore, L1 is involved in modification of synaptic efficacy both *in vitro* and *in vivo* as well as in learning and memory [11], [12]. L1 also promotes myelination in the central and peripheral nervous systems [13], [14], [15], [16], and thus adds to functional recovery after lesioned axons have re-grown. L1 also acts via heterophilic binding mechanisms [17], [18], [19], [20], [21], [22].

In previous studies, it has been shown that soluble, recombinantly expressed dimeric L1 containing the extracellular domain of the molecule in fusion with the Fc portion of human immunoglobulin (L1-Fc) promotes locomotor recovery in adult rats after contusion spinal cord injury [2], [15]. Furthermore, retinal ganglion cell axons regrow in an L1-Fc conducive environment after optic nerve transection [3], [16]. Embryonic stem cells transfected to overexpress full-length L1 at the cell surface support regrowth and reduce dying-back of corticospinal tract axons after spinal cord injury in adult mice, in addition to enhancing survival of L1 overexpressing stem cells versus non-overexpressing stem cells [23]. Enhanced functional recovery and regrowth of serotonergic axons from the brain, reduced dying-back of corticospinal tract axons, reduced expression of the neurite outgrowth inhibitory chondroitin sulfate proteoglycan glycan NG2, and reduced expression of the astrogliosis driven increase of glial fibrillary acidic protein were observed [1]. Furthermore, L1-mediated changes in signal transduction pathways of regrowing axons and/or cells resident in the lesioned spinal cord showed a pronounced influence of L1 on growth-related molecules [24]. The combined results have encouraged further use of L1 in lesion paradigms, not only relating to spinal cord injury, but also in other types of central nervous system trauma [23], [25], [26], [27] and peripheral nerve injury [28].

Given the potential of L1 to be of therapeutic value in nervous system diseases and that its homophilic interactions are conducive, it deemed important to improve L1's efficacy in injuries of the central nervous system. We hypothesized that secretion of a trimer presenting its extracellular domain by stem cells constitutively overexpressing full-length L1 at the cell surface under the control of a universal promoter would enhance regeneration after spinal cord injury even more so than the L1 overexpressing cells. A trimer of the extracellular domain of L1 was constructed for expression with the aim to enhance L1's capacity to cluster cell surface expressed full-length L1 via homophilic interactions, thus inducing signal transduction events conducive to neurite outgrowth and neuronal survival and conditioning the cellular environment of the injured host spinal cord to enrich the hostile host tissue with a regeneration-conducive adhesion molecule. Here we report that the thus engineered stem cells are more potent in recovery of locomotor functions than the parental cells not expressing L1 or overexpressing full-length L1.

## Materials and Methods

### Generation and characterization of embryonic stem cells stably secreting the trimerized extracellular domain of L1

The construct p901-L1-ccCMP-His6 was generated using pECE L1-ccCMP-His6 as a template which was kindly provided by Dr. Heike Hall (Swiss Federal Institute of Technology Zuerich). In this construct, the mouse L1 extracellular domain (amino acid residues 1 to 1,131) is connected at the carboxy-terminus with a sequence encoding the cartilage matrix protein's (CMP's) coiled-coil domain (CMPcc) to generate L1 homotrimers by covalently assembling the CMPcc domains [29]. Considering the necessity of antibiotic selection after transfection into the parent embryonic stem cell (ESC) line overexpressing full-length L1, which was constructed with neomycin resistance, the trimer encoding sequence was subcloned into the pcDNA3.1/Zeo vector by PCR for zoecin antibiotic selection. The phosphoglycerokinase (PGK) promoter was also introduced. Sequence identity of the construct and secretion of L1 trimer was confirmed by DNA sequencing and transfection of CHO cells followed by Western blot analysis of the culture medium collected as the supernatant of the transfected cells two days after subculture. ESCs constitutively expressing full-length L1 [30] were then transfected with this construct and two stable ESC lines were established. The ESC line, the ESC line overexpressing full-length L1 and the ESC cell line overexpressing full-length L1 and secreting the L1 trimer (hereafter designated wt ESCs, L1 ESCs and tL1 ESCs, respectively) were subjected in vitro to the same neural lineage differentiation protocol [31] to generate neural stem cells (NSCs). More than 95% of these cells were positive for nestin as described [25].

To verify secretion of the trimer in full-length L1 overexpressing ESCs two stably transfected ESC lines were maintained for eight days in culture and the supernatants and cell lysates were collected, dialyzed against 10 mM Tris-HCl (pH 7.4) and 150 mM NaCl, and concentrated ∼10-fold in an Amicon Ultra centrifugation filter device (Millipore, Jaffrey, NH). Samples were subjected to 7.5% reducing and non-reducing SDS-PAGE and probed for by Western blot analysis using a polyclonal antibody against full-length mouse L1, kindly provided by Dr. Martin Grumet (Rutgers University). Protein was determined by BCA protein concentration assay kit (Thermo Fisher Scientific, Rockford, IL).

### Animal surgery and cell transplantation

Female C57BL/6J mice, 3 months old, were deeply anaesthetized by intraperitoneal injections of ketamine and xylazine (100 mg of Ketanest and 5 mg of Rompun per kg body weight). Surgery was performed as described [1]. In brief, laminectomy was performed at the T7–T9 level and the spinal cord was lesioned using a compression device which controls compression force (degree of closure of the forceps) and duration of compression. The spinal cord was maximally compressed (100%, according to the operational definition of Curtis and colleagues [32], [33]) for 1 s by a time-controlled current flow through the device. One µl of tL1, L1 and wt NSCs (1×10^5^ cells in phosphate-buffered saline, pH 7.3 (PBS)) was injected 0.5 mm both rostral and caudal to the lesion site using a glass micropipette (tip diameter 100 µm) 1 mm deep into the spinal cord [23]. The locomotor behavior was assessed weekly after the injury until six weeks. Mice were then sacrificed for histological and biochemical analyses in a blinded manner. All surgical procedures and post-operative care were approved by the Rutgers University Animal Care Committee (permit number: 95051) following the guidelines of the National Institutes of Health.

### Evaluation of locomotor behavior

Ground locomotion was evaluated every week from 1 week to 6 weeks after injury using the Basso Mouse Score (BMS) rating scale [34]. Twelve mice evaluated for locomotor activity before surgery were randomly assigned to each group that received an intraspinal injection of tL1, L1, wt NSCs, or PBS only. In addition to BMS assessment, locomotor recovery was analyzed using a single-frame motion analysis including evaluation of three parameters in two different tests: a beam walking test (foot-stepping angle and rump-height index) and a test for voluntary movements without body weight support (extension–flexion ratio) [35]. In addition to analyzing the values for these parameters, recovery indices were calculated [35]. The recovery index (RI) is an individual animal estimate for any given parameter described above and is calculated in percent as: RI = [(X_7+n_−X_7_)/(X_0_−X_7_)]×100, where X_0_, X_7_ and X_7+n_ are experimental values prior to injury, 7 days after injury, and a time-point n days after injury, respectively. Values for the left and right extremities were averaged. Only animals that showed a foot-stepping angle of 160° or more 7 days after injury were considered severely lesioned. All other animals were excluded from further analysis. One animal each was excluded from the tL1 NSC group and PBS vehicle only group, respectively.

### Animal perfusion and immunocytochemistry

Animals were deeply anesthetized by intraperitoneal injection of ketamine and xylazine (100 mg of Ketanest and 5 mg of Rompun per kg body weight) and perfused transcardially with cold phosphate buffer, pH 7.4 (0.1 M) followed by 4% paraformaldehyde in 0.1 M phosphate buffer. A 3 cm long tissue piece of the spinal cord centered at the lesion site was dissected out and processed for the different types of analysis as follows.

For cryotomy, the spinal cords were post-fixed in the perfusion solution plus 10% sucrose overnight at 4°C, and then cryoprotected in 20% sucrose in PBS for 48 h at 4°C. Then, the spinal cord centered at the lesion site was dissected and embedded in mounting media (HistoPrep, Fisher Scientific, Pittsburgh, PA) on dry ice. Cryostat sections (30 µm thick) were cut and stored at −70°C.

For immunohistochemistry, sections were permeabilized with 0.1% (w/v) Triton X-100 in PBS for 20 min at room temperature. Non-specific adsorption was minimized by incubating the sections in 2% (v/v) normal goat serum in PBS for 20 min at room temperature. Sections were incubated in the blocking solution with primary antibody at 4°C overnight. For control, non-immune mouse IgM and IgG as well as rabbit IgG were used as primary antibodies. Slides were washed three times with PBS and incubated with Alexa 555-conjugated goat anti-mouse IgG or Alexa 555-conjugated goat anti-rabbit IgG (1∶800, Jackson Immunoresearch, West Grove, PA) for 2 h at room temperature. Sections were then washed with PBS, and some sections were incubated with 4′-6-diamidino-2-phenylindole (DAPI) for 10 minutes, rewashed with PBS, mounted with Aqua Poly/Mount medium (Polysciences, Warrington, PA), and imaged with an Axiovert200 Fluorescence Live Cell Imaging Workstation (Zeiss). Control sections did not yield detectable signals.

### Estimation of lesion volume

Lesion volume was estimated in parasagittal sections spaced 200 µm apart and stained with polyclonal antibodies against GFAP (Dako, Carpinteria, CA, USA) and fibronectin (1∶500, Sigma) to estimate the scar volume using the Cavalieri principle.

### Quantification of serotonergic axons

Parasagittal sections were double-immunostained for serotonin (5-HT) (1∶400, Abcam, Cambridge, MA) and fibronectin (1∶500, Sigma, St. Louis, MI) to determine 5-HT+ fibers which had sprouted/regrown into lesion site and caudal beyond the fibronectin-immunostained lesion site 6 weeks after the injury. 5-HT+ fibers were imaged with the Axiovert 200 Fluorescence Live Cell Imaging Workstation (Zeiss) and quantified by AxioVision software (Zeiss). Background intensity from an area with no 5-HT immunoreactivity was subtracted from the intensity value. The values are presented as the area of 5-HT+ fibers from 2 mm rostral to the lesion site and the % ratio of fluorescence measured 1 mm caudal to the lesion over the background immunofluorescence measured rostral (1 mm) to the lesion site, respectively.

### Quantification of axon density in the corticospinal tract

For the assessment of axons in the corticospinal tract (CST) by PKC-γ immunostaining, free-floating cross-sections (50 µm thick) were blocked and then incubated with anti-PKC-γ antibody (1∶200, Santa Cruz Biotechnology, Santa Cruz, CA) overnight at 4°C. After washes in PBS, the sections were incubated with fluorescent Alexa 555 goat-anti mouse secondary antibody (1∶800, Invitrogen). Images were taken of the dorsal spinal cord columns at 10×magnification using Axiovert 200 Fluorescence Live Cell Imaging Workstation (Zeiss) at 3, 6, 9, and 11 mm rostral to the injury center. Using ImageJ Software (NIH), the relative intensity of PKC-γ immunoreactivity was measured in a traced area within the dorsal columns. Background intensity from an area with no PKC-γ immunoreactivity was subtracted from the intensity value. Automatic thresholding for each image using ImageJ software was performed to determine the threshold for specific immunoreactivity. After setting the threshold, the immunostaining intensities above the threshold were quantified.

### Western blot analysis

Spinal cords were dissected, placed in ice-cold artificial CSF (124 m*M* NaCl, 3 m*M* KCl, 1 m*M* NaHPO4, 26 m*M* NaHCO3, 1.5 m*M* MgSO4, 1.5 m*M* CaCl2, and 10 m*M* glucose pH 7.0), and cleaned from meninges and nerve roots. A 1 cm long tissue piece of the cord centered at the lesion site was dissected and homogenized (in 50 mM Tris-HCl, pH 7.4, 1 mm EDTA, 0.1% SDS, 100 µm leupeptin, 1 µm pepstatin, 10 µg/ml aprotinin, and 100 µ*M* phenylmethylsulfonyl fluoride). For slot blotting, 100 µg of total protein/well was transferred to polyvinylidene difluoride membranes using a Bio-Dot slot blot apparatus (Bio-Rad, Hercules, CA). The membranes were then incubated with rat L1 monoclonal antibody (555) (1∶5000, R&D Systems, Minneapolis, MN), mouse monoclonal chondroitin sulfate antibody (CS56) (1∶500, Sigma-Aldrich, St. Louis, MO), mouse monoclonal NG2 antibody (1∶1000, Abcam, Cambridge, MA), rabbit polyclonal glial fibrillary acidic protein (GFAP) antibodies (1∶200, Sigma-Aldrich, St. Louis, MO), rat monoclonal myelin basic protein antibody (1∶1000, Abcam, Cambridge, MA), mouse monoclonal neurofilament H (NF200) antibody (1∶500 Millipore, Temecula, CA), mouse monoclonal beta-III tubulin antibody (1∶1000, Covance, Emeryville, CA), rabbit polyclonal numb antibody (1∶50, developed by Dr. Catherine Saner and obtained from the Developmental Studies Hybridroma Bank, University of Iowa, Iowa City, IA) and mouse monoclonal glyceraldehyde-3-phosphate dehydrogenase (GAPDH) antibody (1∶5000, Chemicon, Temecula, CA). Secondary mouse, rat or rabbit antibodies conjugated to horseradish peroxidase with ECL illuminescence intensification (Thermo Fisher Scientific, Rockford, IL) were used for detection. The grey value of each band was measured and normalized to the grey value of the corresponding GAPDH band using Gel-Pro Analyzer software (Media Cybernetics, Bethesda, MD).

### Statistical analysis

All numerical data are presented as group mean values with standard error of mean (SEM). Statistical analyses for locomotor behavior were performed by one-way repeated-measures ANOVA comparing groups followed by Tukey's *post-hoc* test. The statistical significance of differences for mean immunoreactivity intensities, grey values of bands in Western blots and ELISA for each group was estimated by analysis of variance (one-way ANOVA), followed by Tukey's *post-hoc* test. The *p*-values<0.05 were considered statistically significant.

## Results

### Identification of secreted trimeric L1 in stably transfected embryonic stem cells

**Figure 1:**
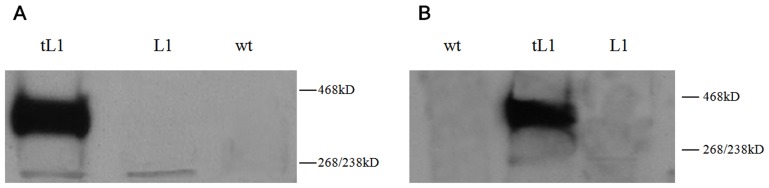
Western blot analysis of L1 as full-length transmembrane molecule and as secreted trimer. (**A**) Identification of trimeric L1 in the lysate of cultured embryonic stem cells under non-reducing conditions. (**B**) Identification of trimeric L1 in the supernatant of cultured embryonic stem cells under non-reducing conditions. wt: parental (wild type) embryonic stem cell line (ESC). L1: ESC line overexpressing full-length L1. tL: ESC cell line overexpressing full-length L1 and secreting the L1 trimer.

Western blot analysis was performed to test L1 as full-length transmembrane molecule and as secreted trimer in cultured embryonic stem cells (ESCs). The expression and secretion of the trimeric L1 was observed in the cell lysate and supernatant of a stably transfected ESC line overexpressing trimeric L1 (tL1) with an apparent molecular mass of 380 kD in SDS-PAGE under non-reducing conditions (**Fig. 1A, B**). The results indicate that the trimeric L1 is secreted.

### tL1 NSCs improve locomotor function better than L1 NSCs and wt NSCs after transplantation into the lesioned spinal cord

**Figure 2:**
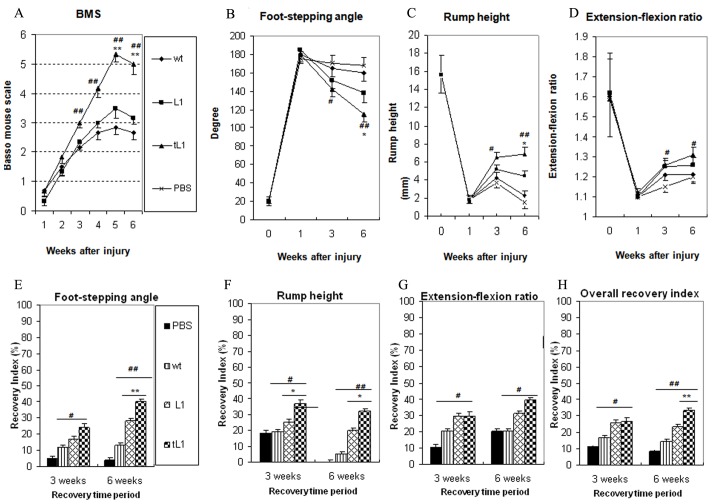
Transplantation of tL1 NSCs improves locomotor recovery after spinal cord injury. Time course and degree of functional recovery after spinal cord injury in mice grafted with tL1 NSCs (n = 11), L1 NSCs (n = 12), wt NSCs (n = 12), or sham-injected with PBS (n = 11). Shown are mean values with SEM as measured by the Basso Mouse Scale (**A**), foot stepping angle (**B**), rump-height index (**C**), and extension-flexion ratio (**D**) one to six weeks after injury. Recovery indices (**E**, **F**, **G**), as shown by mean values with SEM, were calculated from these data. In (**H**) the overall recovery index six weeks after injury is shown. Statistical analysis was performed by one-way ANOVA followed by Tukey's *post-hoc* test. Mice transplanted with tL1 NSCs show better recovery when compared to mice transplanted with L1 NSCs at the time points indicated (* *p*<0.05, ** *p*<0.01) or when compared to mice transplanted with wt NSCs (^#^ *p*<0.05, ^##^ *p*<0.01). (Overall recovery index = [(X_7+n_−X_7_)/(X_0_−X_7_)]×100, where X_0_, X_7_ and X_7+n_ are values prior to injury, seven days after injury, and a time-point n days after injury, respectively).

Spinal cord compression injury caused severe locomotor disabilities in all four experimental groups as estimated by the Basso Mouse Scale (BMS) score one week after injury (**Fig. 2A**). Between 3 and 6 weeks after injury, mice transplanted with tL1 NSCs showed increased mean BMS score values when compared to the other groups transplanted with L1 NSCs at 5 and 6 weeks (*p*<0.01) or wt NSCs at 3, 4, 5 and 6 weeks (*p*<0.01) at the different time points indicating superior locomotor recovery with the maximal mean score values being 5.1±0.16 at 5 weeks (**Fig. 2a**).

We also analyzed the plantar stepping ability of the animals by measuring the foot-stepping angle which was in agreement with the BMS scores. Superior locomotor recovery was observed in mice transplanted with tL1 NSCs when compared to the mice transplanted with L1 NSCs at 6 weeks (*p*<0.01) or wt NSCs at 3 weeks (*p*<0.05) and at 6 weeks (*p*<0.01) (**Fig. 2B, E**). As the foot-stepping angle is a measure of involuntary movement rather than of more complex motor functions, the rump-height index, a parameter to estimate the ability to support body weight during ground locomotion, was analyzed (**Figs. 2C,F**). The results show that mice transplanted with tL1 NSCs had a higher rump-height index and smaller foot-stepping angle than the mice transplanted with L1 NSCs at 6 weeks (*p*<0.05) or wt NSCs at 3 weeks (*p*<0.05) and at 6 weeks (*p*<0.01) (**Fig. 2C, F**). Moreover, the extension-flexion ratio, a parameter to judge voluntary movements without body weight support, showed improvement in the mice transplanted with tL1 NSCs when compared to mice transplanted with wt NSCs at 3 and 6 weeks (*p*<0.05). No differences were observed between the mice transplanted with tL1 NSCs or L1 NSCs, although the former showed a tendency towards improved motor function than the latter three and six weeks after injury (**Fig. 2D, G**). Based on two independent measures, these results indicate that transplantation with tL1 NSCs improves the abilities for ground locomotion after spinal cord injury. A good association between the BMS score and the foot-stepping angle has been described previously [1], [26], [35]. Overall recovery indices for each animal revealed a better overall outcome in mice transplanted with tL1 NSCs compared to mice transplanted with L1 NSCs (*p*<0.01) or wt NSCs (*p*<0.01) at six weeks after transplantation (**Fig. 2H**).

### tL1 NSCs reduce the lesion volume

**Figure 3:**
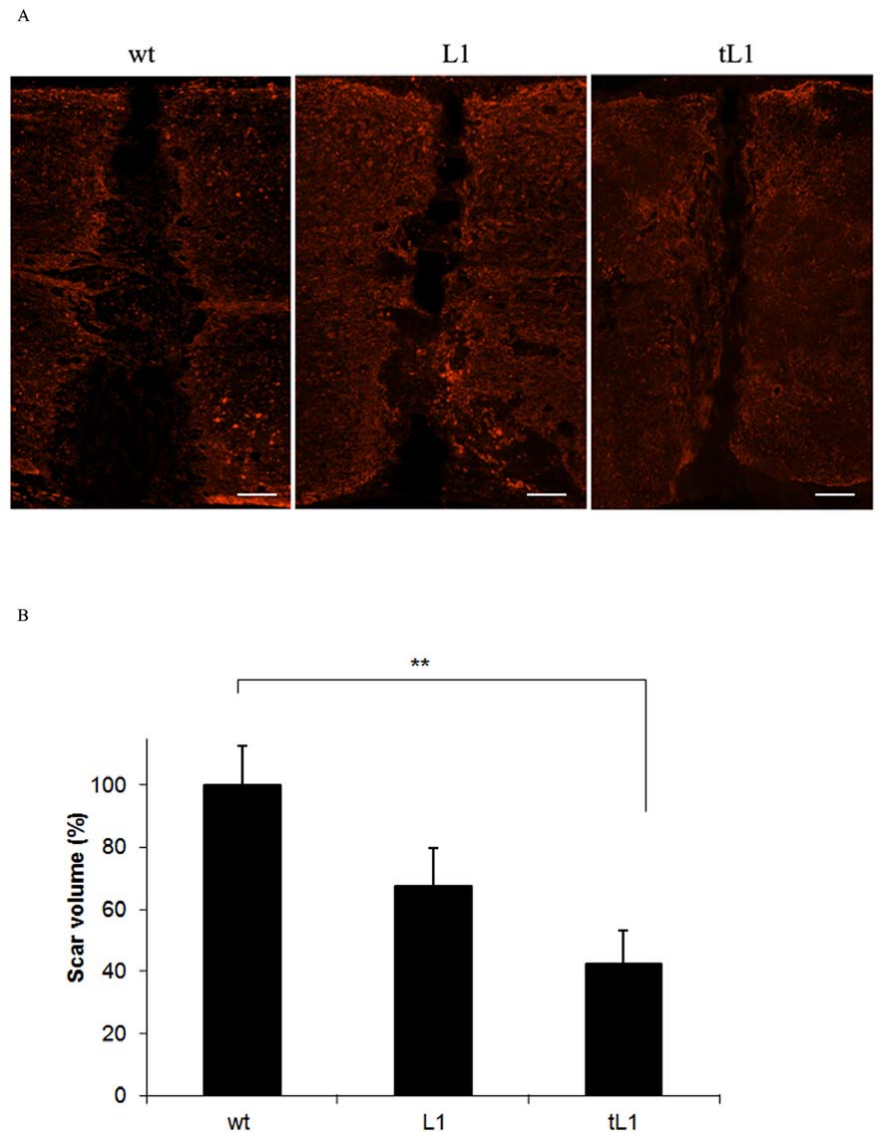
Transplantation of tL1 NSCs reduces lesion volume after spinal cord injury. The lesion sites are delineated by glial fibrillary acidic protein (GFAP) expressing astrocytes (red) six weeks after transplantation of tL1, L1 or wt NSCs. Five mice per group were analyzed. Shown are mean values with SEM from Image J. The scar volume in the wt NSC group was set to 100%. Statistical analysis was performed by one-way ANOVA with Tukey's *post-hoc* test (** *p*<0.01). Scale bar = 150 µm.

Six weeks after transplantation, the lesion area was compared between mice transplanted with tL1 NSCs, L1 NSCs or wt NSCs (**Fig. 3**). Mice grafted with tL1 NSCs showed a reduced lesion volume when compared to mice grafted with wt NSCs (*p*<0.01) and showed a slightly, although not significantly reduced lesion volume in comparison with mice grafted with L1 NSCs (**Fig. 3**).

### tL1 NSC transplantation promotes regrowth/sprouting of serotonergic (5-HT+) axons into and across the lesion site

**Figure 4:**
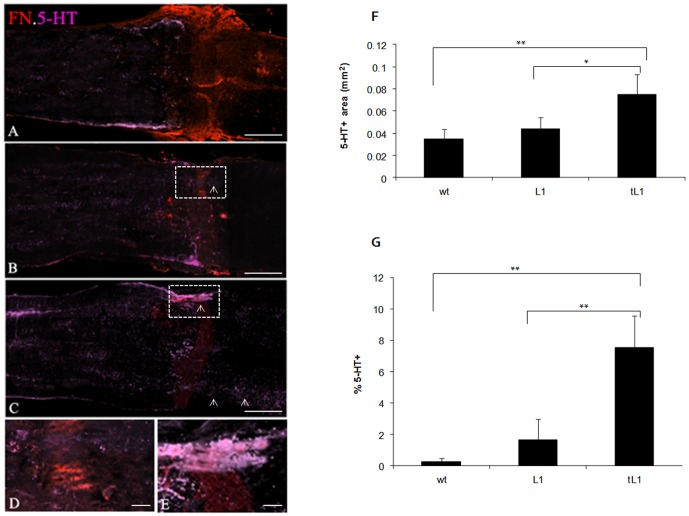
Transplantion of tL1 NSCs promotes regrowth/sprouting of (5-HT+) serotonergic fibers. More 5-HT+ fibers are seen by immunohistology in the areas 2 mm rostral to the lesion site and beyond the caudal border of the lesion site (indicated by fibronectin (FN) immunostaining) in parasagittal sections of mice transplanted with tL1 (**C**, **E**) at 6 weeks after transplantation when compared to mice grafted with L1 (**B**, **D**) or wt (**A**) NSCs. The 5-HT+ fibers which had penetrated the caudal border of the lesion sites are demarcated by the boxes and indicated by arrows. Quantification of 5-HT+ fiber regrowth/sprouting rostral to the lesion site and beyond the caudal edge of the lesion site 6 weeks after injury as determined by AxioVision software analysis (**F**). Values represent the 5-HT+ area at 2 mm rostral to the lesion site (**F**) and the % ratio of fluorescence measured 1 mm caudal to the lesion site over the fluorescence measured rostrally (1 mm) to the lesion site (**G**), analyzed by AxioVision software. Six mice per group were analyzed. Shown are mean values with SEM. Asterisks indicate differences between mice transplanted with tL1 NSCs compared to mice transplanted with wt NSCs or L1 NSCs as assessed by one-way ANOVA followed by Tukey's *post-hoc* test (* *p*<0.05, ** *p*<0.01). Scale bars = 500 µm (**A**, **B** and **C**), 20 µm (**D** and **E**).

5-HT immunoreactive fibers were quantified in the areas 2 mm rostral to the lesion site in parasagittal sections. The results showed higher values in mice grafted with tL1 NSCs in comparison to mice grafted with L1 NSCs (*p*<0.05) or wt NSCs (*p*<0.01) (**Fig. 4F**). It was further examined whether 5-HT+ fibers entered the lesion site and passed beyond the site which was demarcated by fibronectin immunolabeling (**Fig. 4**). 5-HT+ profiles at the caudal border of the fibronectin-immunoreactive lesion site were observed in five out of six mice grafted with tL1 NSCs (**Fig. 4C, E**), only in three out of six mice grafted with L1 NSCs (**Fig. 4B, D**), but none in an animal grafted with wt NSCs (**Fig. 4A**). Quantification of 5-HT+ fibers that had penetrated beyond the caudal border of the lesion site, expressed as a % ratio of the 5-HT+ immunofluorescence intensity measured caudally to the lesion over the fluorescence measured rostrally (1 mm). The ratio showed a difference in mice grafted with tL1 NSCs when compared to the mice grafted with L1 NSCs (*p*<0.01) or the mice grafted with wt NSCs (*p*<0.01) (**Fig. 4G**).

### tL1 NSCs prevent demyelination and/or promote remyelination after spinal cord injury

**Figure 5:**
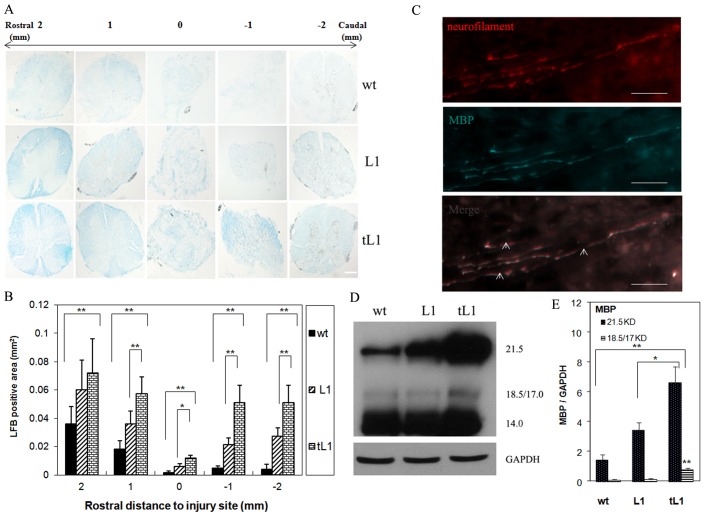
Transplantation of tL1 NSCs prevents demyelination and/or enhances remyelination after spinal cord injury. (**A**) Representative Luxol Fast Blue (LFB) staining images of the cross-sectioned spinal cord 1 and 2 mm rostral or caudal to the lesion site (indicated by 0) from mice transplanted with tL1, L1 or wt NSCs 6 weeks after injury. (**B**) Quantitative analysis of the myelinated area in LFB-stained sections shown in (**A**). (**C**) Representative immunostaining images with antibodies against neurofilament and myelin basic protein (MBP). Neurofilament (NF) -immunoreactive fibers 1 mm rostral to the lesion site are myelinated as shown by colocalization with immunostaining for MBP (indicated by arrows) in the mouse spinal cord transplanted with tL1 NSCs. (**D**) Western blot analysis of of MBP isoforms in homogenates of spinal cord containing the lesion site and 0.5 cm tissue caudal and rostral to the lesion site. (**B**) (**E**) Shown are mean values with SEM of the LBF positive area (**B**) and of MBP expression levels normalized to GAPDH (**E**). Five mice per group were analyzed. Statistical analysis was performed by one-way ANOVA with Tukey's *post-hoc* test (* *p*<0.05, ** *p*<0.01). Scale bars = 200 µm (**A**) and 10 µm (**C**).

To monitor the de-/remyelination resulting from tL1 NSCs transplantion, Luxol Fast Blue staining was performed on cross-sections. Mice transplanted with tL1 NSCs showed larger myelinated areas at the lesion site and 1 or 2 mm rostral and caudal to the lesion site, when compared to the group of mice transplanted with L1 NSCs or wt NSCs (**Fig. 5A, B**). Also more neurofilament immunoreactive fibers were myelinated in the area rostral to the epicenter of the injury, as shown by colocalization with staining of myelin basic protein (MBP) in mice transplanted with tL1 NSCs (**Fig. 5C**). Quantitative evaluation of MBP expression by Western blot analysis showed that expression of the two larger MBP isoforms (21.5 kDa and 18.5 kDa) was upregulated in mice grafted with tL1 NSCs, when compared to mice grafted with L1 NSCs (*p*<0.05 for 21.5 kDa) and (*p*<0.01 for 18 kDa) or wt NSCs (*p*<0.01 for 21.5 kDa and 18 kDa) (**Fig. 5D, E**). No differences between the groups were found for the other MBP isoforms (17.0 and 14.0 kDa) (**Fig. 5D**). Both, Luxol Fast Blue staining and MBP expression indicate that tL1 NSCs prevent demyelination and/or promote remyelination.

### tL1 NSCs promote axonal preservation and/or regrowth/sprouting of corticospinal tract axons after injury

**Figure 6:**
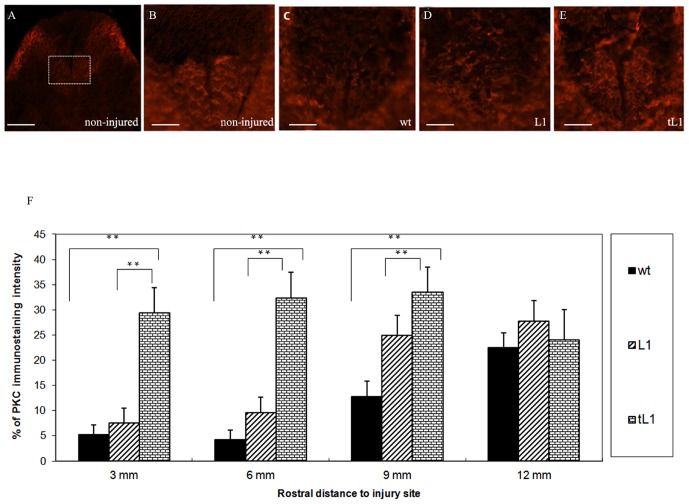
Transplantation of tL1 NSCs promotes regrowth/sprouting of corticospinal tract (CST) axons. (**A–E**) Representative PKC-γ immunostaining images from cross-sections of spinal cord at midthoracic level (6 mm rostral to the lesion site) show localization of CST axons in the ventral part of the dorsal columns of non-injured mice and injured mice transplanted with wt, L1 and tL1 NSCs 6 weeks after injury. The cross-sectional area occupied by CST axons is demarcated by the inset in (**A**) shown at higher magnification in (**B**–**E**). (**F**) Image J quantification of the dorsal column area at 3, 6 and 9 mm rostral to the lesion site shows an increase in PKC-γ immunoreactivity in mice transplanted with tL1 NSCs compared to mice transplanted with wt NSCs or L1 NSCs. Values represent % PKC-γ immunoreactivity normalized to the immunoreactivity from the dorsal columns of non-injured mice. Five mice were analyzed per group. Shown are mean values with SEM. Asterisks indicate differences between mice transplanted with tL1 NSCs compared to mice transplanted with wt NSCs or L1 NSCs as assessed by one-way ANOVA followed by Tukey's *post-hoc* test (* *p*<0.05, ** *p*<0.01). Scale bars = 100 µm (**A**) and 40 µm (**B–E**).

To investigate whether grafting of tL1 NSCs would promote axonal sparing and/or regrowth/sprouting, the integrity of the corticospinal tract (CST) was analyzed. Axons caudal to the lesion site were observed to undergo Wallerian degeneration, whereas axons in the rostral segment were observed to undergo retrograde axonal degeneration (“dying back”). Integrity of the CST was assessed by immunostaining for PKC-γ [36], [37], which specifically marks the main CST at the base of the dorsal columns of uninjured mice (**Fig. 6**). No PKC-γ immunoreactivity was observed in the dorsal columns 3 mm caudal to the lesion site at six weeks after injury. PKC-γ immunostaining intensities were also decreased at the base of the dorsal column at rostral distances between 3 and 12 mm in parasagittal sections compared to the uninjured spinal cord, suggesting axonal degeneration in the rostral part of the CST (**Fig. 6A–E**). Injured CST fibers frequently appeared swollen, indicating dystrophic, “dying back” axonal terminals. All injured mice showed a reduction in intensity of the PKC-γ immunostaining rostral to the lesion in comparison to the uninjured animals (**Fig. 6A–F**). This reduction was more pronounced in mice grafted with wt NSCs or L1 NSCs than in mice grafted with tL1 NSCs (**Fig. 6E, F**). Higher intensities of immunostaining for PKC-γ was seen rostral to the lesion site in mice grafted with tL1 NSCs than in mice grafted with L1 NSCs at 3, 6 mm (*p*<0.01) and 9 mm (*p*<0.05) (**Fig. 6D, F**) or wt NSCs at 3, 6 and 9 mm (*p*<0.01) (**Fig. 6C, F**).

### Western blot analysis of neurite outgrowth inhibitory and beneficial molecules

**Figure 7:**
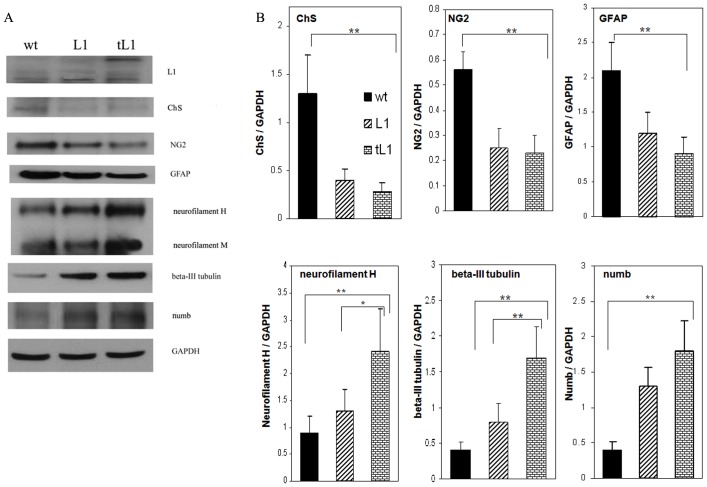
Western blot analysis of neurite outgrowth inhibitory and beneficial molecules in the spinal cords. (**A**) Representative images of Western blots of L1, chrondroitin sulfate (ChS), NG2, GFAP, neurofilament, beta-III tubulin, numb and GAPDH in 1 cm long tissue piece of the spinal cords centered at the lesion site of mice transplanted with wt, L1 and tL1 NSCs 1 week (for L1) or 6 weeks (for the other antigens) after injury. (**B**) Gel-Pro Analyzer software quantified mean values with SEM of protein expression levels normalized to GAPDH. Five mice were analyzed per group. Asterisks indicate differences between mice transplanted with tL1 NSCs compared to mice transplanted with wt NSCs or L1 NSCs as assessed by one-way ANOVA followed by Tukey's *post-hoc* test (* *p*<0.05, ** *p*<0.01).

Western blot analysis under non-reducing conditions identified tL1 in homogenates from 1 cm long spinal cord segments containing the lesion site one week after transplantation with tL1 NSCs. The homogenates from these segments obtained 6 weeks after the injury and NSCs transplantation were probed by Western blot analysis using antibodies against chondroitin sulfate (ChS), GFAP and the chondroitin sulfate proteoglycan (CSPG) NG2. Reduction in the levels of CSPGs, GFAP and NG2 expression was observed in mice grafted with tL1 NSCs, when compared to mice grafted with wt NSCs (*p*<0.01) (**Fig. 7**). Analysis of axon–related molecules showed higher expression levels of both neurofilament H and beta-III tubulin in mice grafted with tL1 NSCs, when compared to mice grafted with L1 NSCs or wt NSCs (**Fig. 7**). The expression levels of the cell fate determinant factor numb which had been found to improve neurite outgrowth by promoting L1 endocytosis at growth cones [38] were higher in mice grafted with tL1 NSCs than in mice grafted with wt NSCs (*p*<0.01).

## Discussion

The present study was undertaken to probe the need for improving the efficacy of the functions of L1 by engineering stem cells to overexpress full-length L1 and, in addition, a secreting the trimer of the extracellular domain of L1 in order to optimize not only for the beneficial homophilic and also heterophilic interactions benefitting the transplanted stem cells in a fashion that could be perceived to act via “autocrine” mechanisms (L1 interacts with itself in trans-interaction), but also to favourably condition the hostile tissue environment in a “paracrine” fashion to tip the balance towards regeneration-conducive cues. The hypothesis was that stem cells with this configuration of L1 expression would not only be stimulated in L1 signal transduction mechanisms leading to a combination of beneficial events in the full-length L1 overexpressing stem cells triggered by the L1 trimer, but also lead to diffusion of the trimeric L1 away from the engineered cells into the host tissue and thus condition it rostral and distal to the lesion site for tissue preservation and overcoming the inhibitory cellular environment of the host, respectively. An example of this conducive effect is that the transmembrane form of L1 is able to overcome its inhibitory signaling complex with neuropilin-1 in vitro by addition of L1-Fc [39].

Our study lends support to the view that we have optimized the beneficial functions of L1 in neural stem cells. It is noteworthy in this context that L1 not only interacts via homophilic mechanisms, but has many ways of interacting in heterophilic modes, one of which relates to its heterophilic cis-interactions resulting in neurite outgrowth and homophilic trans-interactions which results in neuronal survival, via distinct, but also more down-stream similar signal transduction cascades [17]. Also the differentiation of neural stem cells into neurons is enhanced by L1 in a trans-interaction that are likely to involve homophilic and heterophilic interactions in cis and trans [40]. A distinction between hemophilic and heterophilic interaction mechanisms of the secreted L1 is not possible in the complex molecular and cellular context of an injured tissue.

We have shown previously that full-length L1 overexpressing embryonic stem cells differentiated into the neural lineage are able, without trimer expression, to promote murine corticospinal tract axons regrowing into the lesion site of spinal cord and, in some cases, extending beyond it one month after transplantation. These results indicated that L1 expressed by stem cells may confer beneficial properties to the stem cells themselves by presenting a favorable substrate to the severed axons so that they would cross the lesion site and lend to them trophic support [23]. Homophilic interaction of these stem cells were most likely not optimal since the adult host tissue expresses low levels of L1 which is most highly expressed during ontogenetic development of the nervous system. It is noteworthy in this respect that astrocytes and oligodendrocytes do not express detectable levels of L1, whereas the regeneration-conducive Schwann cells express L1 when they de-differentiate into proliferating Schwann cells and differentiate to initiate L1-mediated remyelination after nerve transection caudal to the lesion site of peripheral nerves, thus contributing to successful regrowth and myelination of severed axons. Based on these findings we engineered astrocytes - in addition to neurons - to express L1 via adeno-associated viral transduction and found that this manipulation enhanced spinal cord regeneration after injury [1]. We have also infused L1 in the dimeric form of its extracellular domain into the contusion-lesioned rat spinal cord and observed enhanced functional recovery [2]. The interpretation of this result was that a diffusible L1 molecule conditions the tissue environment such that it overcomes the inhibitory cues of the host such that neuritogenesis is not compromised [41].

Based on these observations we sought to combine the regeneration-promoting effect of transmembrane-anchored L1 with diffusible L1 to promote regeneration after injury. We observed beneficial effects of this combination by assessment of locomotor recovery using the BMS score and quantitative evaluation of hind leg posture indices and, as observed previously [1], [26], [35], found a good correlation between the BMS score and, in particular, the foot-base angle as seen in video-recordings. The locomotor parameters correlated with histological/immunohistological parameters as quantified by glial scar formation - being reduced because of L1-mediated neuronal/axonal protection - and the following parameters: enhanced density of serotonergic fibers in and beyond the glial scar, sparing of axons of the corticospinal tract rostral to the lesion site, and sparing of myelin and/or enhancing remyelination. The size of glial scar was taken into account, since it correlates positively with the extent of the lesion and neuronal/axonal injury. The other parameters were chosen as characteristic features of correlation between locomotor recovery and cellular disposition. The corticospinal tract does not contribute to locomotor recovery, when spinal cord lesions are carried out at the thoracic level, but sparing of these axons is an indicator of protective effects and was thus added to the parameters measuring the influence of L1 in the injured host. It is plausible to expect that other cellular parameters of successful locomotor recovery would be beneficially influenced by the L1 overexpressing stem cells and by soluble L1, but since they have been described before, they were not reiterated in the present study. As in our previous studies, there were no indications that animals suffered from allodynia or hyperalgesia, neither in the mice transplanted with the parental NSCs nor in mice transplanted with L1 NSCs or tL1 NSCs.

The results of the present study encourage the view that neural stem cells can be used to optimize the beneficial functions of L1 and thus contribute to an improved amelioration of the behavioral deficits after injury in the acutely lesioned spinal cord. Based on our previous studies we hope that the optimized cells would also ameliorate the deficits in mouse models of neurodegenerative diseases, such as Huntington's [25], Parkinson's [26] and possibly other nervous system diseases.
